# Rituximab in Childhood and Juvenile Pemphigus Vulgaris: A Systematic Review

**DOI:** 10.7759/cureus.58288

**Published:** 2024-04-15

**Authors:** Priyanshu Shrivastava, Sarah Mariam, Laraib Abid, Sajad A Buch, Syed A Ahmad, Shahnaz Mansoori, Shamimul Hasan

**Affiliations:** 1 Faculty of Dentistry, Jamia Millia Islamia, New Delhi, IND; 2 Periodontology, Bharati Vidyapeeth Deemed To Be University, Pune, IND; 3 Clinical Oral Health Sciences, School of Dentistry, IMU University, Kuala Lumpur, MYS; 4 Oral and Maxillofacial Surgery, Faculty of Dentistry, Jamia Millia Islamia, New Delhi, IND; 5 Oral Medicine and Radiology, Faculty of Dentistry, Jamia Millia Islamia, New Delhi, IND

**Keywords:** corticosteroids, rituximab, autoimmune, pediatric pemphigus vulgaris, pemphigus vulgaris

## Abstract

Pemphigus vulgaris (PV) is a chronic autoimmune blistering disorder characterized by the loss of intraepithelial adhesion, affecting the skin and mucous membranes. Both males and females are affected, although it predominantly affects females in their fifth and sixth decades of life. Approximately 1.4 to 3.7% of PV cases occur in the pediatric population (≤18 years of age), and may be classified into childhood/pediatric PV, which affects individuals under 12 years old, and juvenile/adolescent PV, affecting those between 12 and 18 years old. Due to its rare occurrence in children and adolescents, there is often a delay in diagnosis and treatment in this age group.

A systematic literature search was conducted on MEDLINE/PubMed, Web of Science, EMBASE, SCOPUS, and Cochrane Library databases to evaluate the efficacy of rituximab (RTX) in childhood and juvenile PV patients. The Joanna Briggs Institute (JBI) Critical Appraisal Checklist was employed to assess the risk of bias in case reports and series, while the Cochrane ROBINS-I tool was utilized for evaluating observational studies or non-randomized intervention studies.

A total of 18 studies encompassing 46 juvenile or childhood PV patients in the pediatric and adolescent age groups were included for qualitative synthesis. The studies included nine case reports, two case series, five retrospective studies, one prospective study, and one open-label pilot study. Almost all cases of childhood and juvenile PV achieved either complete or partial remission after undergoing RTX treatment during the final follow-up periods. Furthermore, most cases reported no relapse, and only minor adverse events were noted in the RTX treatment group.

Despite its potential benefits, the utilization of RTX in pediatric patients raises concerns due to the scarcity of evidence and the absence of controlled studies specific to this age group. Further exploration is necessary to establish a standardized treatment regimen for RTX in pediatric PV, which involves identifying the optimal dosage, frequency, treatment cycle duration, and maintenance therapy duration.

## Introduction and background

Pemphigus refers to a diverse range of chronic blistering conditions that affect both mucous membranes and skin. These disorders are typified by IgG autoantibodies targeting keratinocyte adhesion proteins (desmogleins Dsg1 and Dsg3). The binding of IgG autoantibodies to desmosomal complexes leads to a disruption in intraepidermal adhesion, which causes loss of cell-cell adhesion (acantholysis). This results in the formation of vesicles, blisters, and erosions on the skin and mucous membranes [1].

Pemphigus vulgaris (PV) and pemphigus foliaceus (PF) form the major types of pemphigus. Other less frequently occurring forms include pemphigus vegetans, pemphigus herpetiformis (PH), pemphigus erythematosus, IgA pemphigus, and paraneoplastic pemphigus (PNP). PV is recognized as the most frequently occurring type of pemphigus, accounting for approximately 70% of all cases [2]. Although PV is considered an autoimmune disorder, the specific mechanism of desmosome breakdown after autoantibody binding remains unclear. Multiple theories, such as the steric hindrance theory, desmoglein compensation theory, multiple hits hypothesis, and antibody-induced apoptosis and signaling theory, have been proposed in the literature but have not yielded conclusive results [3]. Additionally, non-Dsg IgG serum autoantibodies have been reported to play a role in pathogenesis. These specific autoantibodies have been identified to target a variety of structural and metabolic proteins, such as desmocollins (Dsc) 1 and 3, muscarinic and nicotinic acetylcholine receptors, mitochondrial antigens, thyroid peroxidase, hSPCA1, plakophilin 3, plakoglobin, and E-cadherin. These non-Dsg autoantibodies might act in synergy with the classical effects of anti-Dsg autoantibodies, thereby contributing to the multifaceted process underlying pemphigus pathogenesis [4]. Various antigenic triggering factors have also been identified that play a role in PV pathogenesis. These include viral infections, genetic factors, thiol group drugs (penicillamine, captopril, and rifampicin), food (such as garlic), vaccines, radiation therapy, pregnancy, micronutrients, and stress [5,6].

The worldwide incidence of PV is 0.1-0.5 per 100,000 people per year; however, this varies from 0.17 per million per year in France to 6.8 per million per year in the United Kingdom. The PV incidence in India ranges from 0.09% to 1.8%. Additionally, PV is more common in Jewish populations, particularly those of Ashkenazi origin, and in the Mediterranean [5,7].

PV exhibits an age and site predilection and typically affects females during their fifth or sixth decade of life [7]. Approximately 1.4% to 3.7% of all PV cases are observed in individuals aged 18 years or younger. PV in the pediatric group can be categorized as childhood/pediatric PV, affecting those under 12 years of age, and juvenile/adolescent PV, affecting individuals between the ages of 12 and 18 years. The majority of pediatric pemphigus cases are of the vulgaris type, generally manifesting at approximately 12 years of age [8].

Pediatric PV cases can pose a diagnostic challenge because of their rarity and are frequently identified only after a more advanced clinical presentation [9]. Due to the limited number of controlled trials in pediatric PV, there are no approved therapeutic protocols by the Food and Drug Administration (FDA), and the existing therapeutic protocols lack substantial evidence. Currently, there are no specific guidelines for therapeutic strategies for this patient population [10,11].

Systemic corticosteroids form the cornerstone therapy for PV, while adjuvant therapies such as mycophenolate mofetil, azathioprine, dapsone, cyclophosphamide, and rituximab (RTX) are used in recalcitrant cases. These current therapies are effective in reducing circulating antibodies and allowing patients to lead their normal lives [12].

RTX, a monoclonal antibody composed of both murine and human elements, targets the CD20 antigen on B lymphocytes. It serves as a crucial therapeutic tool for numerous B-cell malignancies. Its ability to deplete B cells makes it effective in managing various autoimmune disorders where autoantibodies are believed to contribute to the disease process [13]. Originally employed as an off-label agent in pemphigus treatment, rituximab's usage has steadily risen, revolutionizing the management of immunobullous diseases. This shift has moved the emphasis from broad immunosuppression to precise immunotherapy, with rituximab now being recommended as the primary treatment option, particularly for newly diagnosed pemphigus patients [14].

Although the efficacy of RTX in the pediatric population is promising, it lacks evidence due to limited studies conducted. Hence, this systematic review was carried out to evaluate the efficacy of RTX in childhood and juvenile PV patients.

## Review

Protocol and ethics

Preferred Reporting Items for Systematic Review and Meta-Analysis (PRISMA) standards were followed in conducting this systemic review [15]. The research question was defined by PICO as follows: 

Participants (P): Patients under 18 years of age with a confirmatory diagnosis of PV through histopathological or immunofluorescence studies. 

Interventions (I): Rituximab was administered in all forms and dosages.

Comparator (C): There was no control taken due to the scarcity of clinical trials.

Outcomes (O): The outcome measures were based on the consensus statement by Murrel et al. in terms of clinical endpoints [16].

Study design (S): All study designs were included except reviews, personal opinions, conference proceedings, and letters to the editor.

Search strategy

A literature search was carried out by two investigators on databases MEDLINE, PubMed, Web of Science, EMBASE, SCOPUS, and the Cochrane Library database for studies published from inception until December 2023. The Google Scholar search engine was also used to ensure the comprehensiveness of the search and to identify any gray literature. The following keywords were used for the search: "Childhood/Juvenile", "pediatric", "Pemphigus Vulgaris", "Pemphigus", "Rituximab", "AntiCD20", and "immunosuppressants" in different combinations using Boolean operators to yield maximum results. Manual screening of the reference list was also performed to identify studies missed by our electronic search. The following inclusion and exclusion criteria were considered (Table 1).

**Table 1:** Inclusion and exclusion criteria.

Inclusion criteria	Exclusion criteria
Diagnosed cases of pemphigus vulgaris in patients under the age of 18 years treated with rituximab.	Patients above the age of 18 diagnosed with pemphigus vulgaris and other forms of pemphigus.
Randomized controlled trials, prospective or retrospective cohort studies, case-control studies, case series, and case reports published from inception until December 2023.	Reviews, personal opinions, conference proceedings, and letters to the editor.
Studies published in the English language.	Studies published in languages other than English.

Data extraction

Relevant data were extracted by two experienced investigators after shortlisting the final articles. Data about bibliographic information including author details, year of publication, study design, sample size, age group, and sex ratio were extracted, followed by clinical details about the duration of disease, past-treatment history, indications for RTX, treatment protocol, additional treatment requirements, follow-up period and post-interventional outcomes.

Quality assessment

The risk of bias judgment was done manually by two investigators. Joanna Briggs Institute (JBI) Critical Appraisal Checklist was utilized to calculate the risk of bias for case reports and series [17]. Cochrane ROBINS-I tool was used for the assessment of observational studies or non-randomized studies of intervention [18].

Results

Identification of Studies

**Figure 1:**
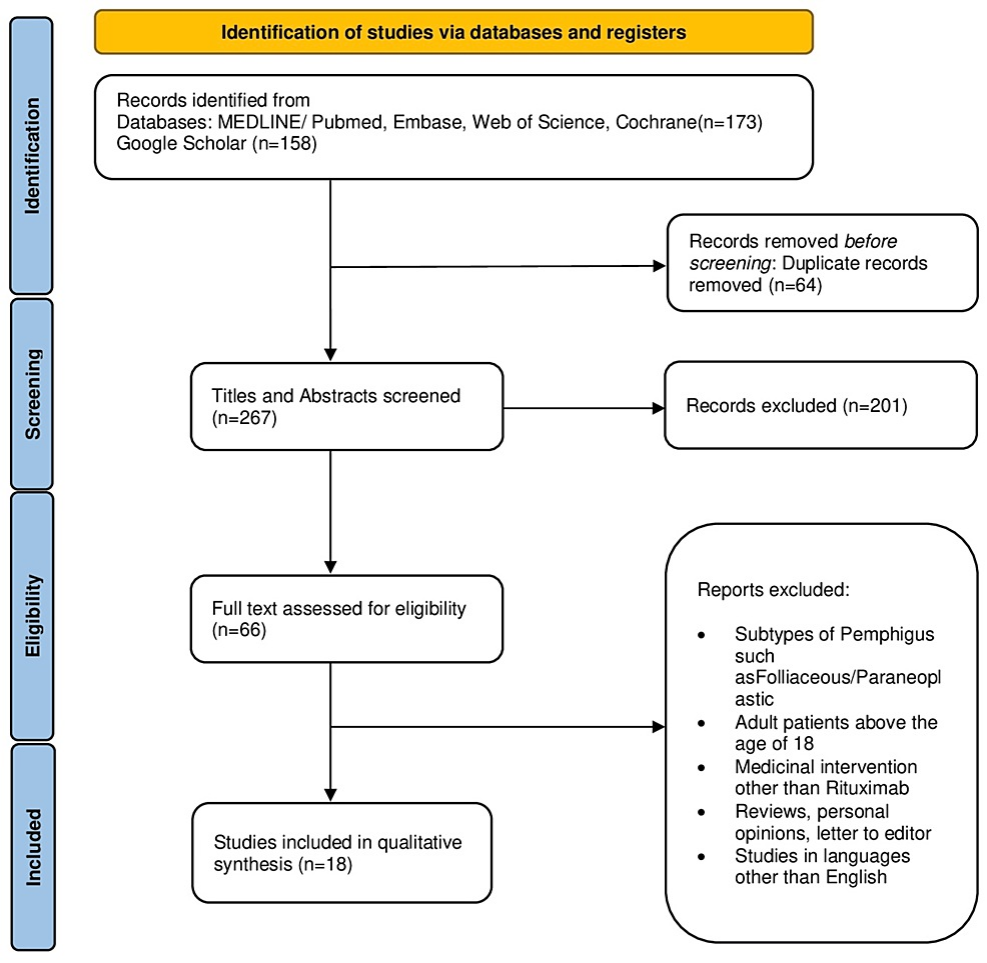
PRISMA flowchart. PRISMA: Preferred Reporting Items for Systematic Reviews and Meta-Analyses

A total of 331 studies were found following an initial search on all databases and the Google Scholar search engine. Of them, 64 duplicates were removed and titles/abstracts of 267 articles were screened. After initial screening, 66 full texts were reviewed by the defined eligibility criteria and PICO as illustrated in Figure 1. 

Study Characteristics

A total of 18 studies were shortlisted for qualitative synthesis [9,11,19-34]. The characteristics of the included studies are comprehensively elaborated in Table 2.

**Table 2:** Detailed characteristics of the included studies. PV: pemphigus vulgaris; F: female; M: male; CR: corticosteroids; +: positive;  IF: immunofluorescence; DIF: direct immunofluorescence; IIF: indirect immunofluorescence; Pred: prednisolone; TCA: triamcinolone acetonide; HC: hydrocortisone; MP: methylprednisolone; BM: betamethasone; DM: dexamethasone; DMP: dexamethasone pulse; MTX: methotrexate; Aza: azathioprine; Dap: dapsone; Cyp: cyclophosphamide; Cys: cyclosporine; MMF: mycophenolate mofetil; IVIG: intravenous immunoglobulin G; ACT: acetaminophen; CPR: chlorpheniramine; CLR: clarithromycin; ACV: acyclovir; AV: aloe vera; RTX: rituximab; TS: Tzanck smear; HP: histopathology; URT: upper respiratory tract infection; NA: not available; NR: not reported; CT: conventional therapy; HZ: herpes zoster; ELISA: enzyme-linked immunosorbent assay.

S.No.	Author, reference	Study design	No. of patients	Age range/mean (years)	Sex	Site	Confirmatory tests	Disease duration before RTX	Past treatment details	Indication for RTX treatment	Current treatment details	Additional treatment required	Follow-up period	Relapse/flare	Final outcome	Adverse events	Conclusion
1	Bilgic‐Temel et al. 2019 [19]	Retrospective analysis	5	15 (11-17)	3 M: 2 F	Mucocutaneous PV with prominent involvement of the oral mucosa.	Biopsy, DIF	26.2 months (9-42)	MP (ranging between 0.5 and 1 mg/kg/daily), Aza, MMF, Dap, and IVIG	CRs adverse effects; persistent old lesions/recalcitrant established lesions	Patients were treated with either a fixed‐dose RTX regimen or a body surface RTX regimen	Additional fixed‐dose RTX regimen infusions given in two patients and the body surface regimen was administered in one.	42.6 months (19-60)	3 out of 5 cases showed relapse	Complete (3) and partial (2) remission achieved off treatment	None	Complete and partial remission achieved with no adverse events
2	Broshtilova et al. 2019 [20]	Case report	1	14	F	Face; trunk and extremities, oral cavity	Biopsy, DIF, IIF	NR	MP 40 mg/day, Dap 25 mg/day	Refractory lesions and CRs adverse effects	RTX therapy 2 doses of 375 mg/m^2^ 30 days apart	Low-dose steroid therapy	34 months	No	Complete remission	NR	RTX is efficient, well-tolerated, and safe in a low dose
3	Buch et al. 2016 [21]	Case series	3	11	F	Skin, scalp, oral lesions	TS and Biopsy	4 months	I.V DM (1 cc) twice a day with gradual tapering along with Aza once a day for 22 days	New lesions despite treatment; Adverse effects of CRs, DM, and Aza	300 mg RTX infusion for 5-6 hours, then a second dose after 15 days.	40 mg Pred	12 months	1 patient relapsed out of 3	Complete remission on treatment (after 4 doses and 1.5 years of therapy)	None	RTX is an effective adjuvant therapy for resistance in pediatric PV
				12	M	Skin, scalp, oral lesions	TS and Biopsy	14 months	I.V DM (1 cc) twice a day with gradual tapering along with Aza once a day for 22 days	New lesions despite treatment; Adverse effects of CRs, DM, and Aza					Complete remission on treatment (after 2 doses and 1.5 years of therapy)		
				9	F	Scalp and oral lesions	TS and Biopsy	6 months	I.V DM (1 cc) twice a day with gradual tapering along with Aza once a day for 22 days	New lesions despite treatment; Adverse effects of CRs					Complete remission on treatment (after 4 doses and 1 year of therapy)		
4	Chen et al. 2013 [22]	Case report	1	17	M	Oral ulcers	Biopsy		IV MP (1.2 mg/kg/day)	1	IV/RTX (500 mg) weekly for four doses	NR	NR	NR	Complete remission off treatment	NR	Steroid is the first-line therapy of PV and RTX is promising in refractory cases or steroid-sparing effect
5	Fuertes et al. 2010 [23]	Case report	1	18 months	M	Skin and oral mucosa	Biopsy, DIF; IIF	14 years	Pulses of MP at 6 mg/kg/day followed by high-dose oral pred; oral plus Cys, Pred, Aza, and Dap	Persistent lesions and adverse CR effects	RTX (375 mg/m^2^ of body surface area) with 4 infusions of RTX at weekly intervals	Concomitant Pred 20 mg/day) rapidly tapered during the next 3 weeks.	16 years	No	Complete remission off treatment	None	RTX is a safe and efficacious therapy for severe pediatric PV
6	Gupta et al. 2015 [24]	Prospective study	5	12	M	NR	Biopsy; DIF	6 months to 10 years	40 mg CRs OD + 50 mg Aza, Cyp	Not responding to any other form of therapy	500 mg RTX over 6 hours, another dose after 2 weeks	CRs (5-20 mg/day)	12 months	No	Complete remission off treatment	Infusion reaction and HZ Infection	Low‐dose RTX used as an adjuvant therapy in pediatric PV with minimal side effects
				9	F							CRs (5-20 mg/day)		No	Complete remission off treatment		
				11	F							Cyp (50 mg/day) CRs (5-20 mg/day)		No	Complete remission on treatment		
				12	M							Cyp (50 mg/day) CRs (5-20 mg/day)		No	Complete remission on treatment		
				9	M							CRs (5-20 mg/day)		No	Complete remission off treatment		
7	Kanwar et al. 2013 [25]	Open-labeled pilot study	1	9	M	Not specified	Biopsy, DIF	6 months	Aza, DMP, Pred	Refractory to CRs; Severe disease	375 mg/m^2^, 2 doses 15 days apart	1.0 mg/kg/day of Pred	46 weeks	NR	Complete remission off treatment	Angioedema	Low‐dose RTX used as an adjuvant therapy in pediatric PV with minimal side effects
8	Kanwar et al. 2012 [26]	Case report	1	11	M	Face and upper trunk	TS, Tzanck smear, biopsy, DIF, IIF	4 months	DMP therapy (100 mg DM in 250 mL of 5% dextrose for 3 days every 28 days); oral Pred 1 mg/kg/day and Aza 1.5 mg/kg	Severe disease flare with extensive cutaneous erosions and blisters	375 mg/m^2^, 2 doses 15 days apart	Pred at a dose of 1 mg/kg/day	8 months	NR	Complete remission of treatment	None	RTX may be an adjunct in recalcitrant pediatric PV
9	Kianfar et al. 2022 [27]	Retrospective, single-center study	10	11-17	3M: 7F	Cutaneous and mucocutaneous involvement	Biopsy, DIF, ELISA	4 months	Pred, Aza, MMF, intralesional CRs	Resistant lesions, side effects of CRs, severe flares of PV	RTX 375 mg/m^2^ weekly (up to 500 mg in each infusion), for four weeks	Oral ACT, I.M CPR, and I.V HC as premedication to prevent infusion reactions	5-103 months	2 major and 5 minor relapses	Complete or partial remission on minimal therapy	Chills, fever, dsypnea, rigor, tachycardia	RTX can be used in moderate-severe PV as first-line therapy.
10	Kincaid et al. 2016 [28]	Case report	1	4	F	Skin, face, oral cavity	Biopsy, DIF	5 days	Pred, CLR, ACV, Aza, and IVIG	Disease progression despite treatment and CR-related adverse effects	375 mg/m^2^ of body surface area, 15 days apart. Received every 4-8 weeks; maintenance = every 8-12 weeks	High-dose Pred and second two-dose RTX cycle therapy resulted in full clinical remission within 2 weeks. CRs and Aza discontinued 2 and 10 months later	2 years	Disease relapsed twice, 3 months and 13 months post-treatment	Complete remission off treatment	Isolated infusion reaction with the second cycle consisting of urticaria and low-grade fever	Two-dose protocol of 375 mg/m^2^ of BSA given 15 days apart was a safe and effective therapy.
11	Kong et al. 2005 [29]	Case report	1	17	F	Skin, oral cavity	Biopsy, DIF	4 years 6 months	Pred (maximum 1.6 mg/kg daily), Aza (1 mg/kg daily), MMF (1 g daily), IVIG (1 g/kg)	Previous treatment failure and continued disease activity	375 mg/m^2^ of body surface area over 5 hours, continued every 4 to 8 weeks	Pred	17 months	No	Complete remission	NR	RTX can be an important therapeutic alternative for pediatric PV unresponsive to CT.
12	Kong et al. 2015 [30]	Retrospective analysis	2	9	M	Face, trunk, upper and lower extremities	IIF Indirect >1/160 intercellular pattern	NR	CRs and adjuvant immunosuppressants	Suboptimal response to previous therapies	375 mg/m^2^, 2 doses 15 days apart	Pred, Aza, MTX, MMF	25 months	NR	Complete remission on therapy	NR	RTX is an emerging effective therapy in recalcitrant pediatric PV.
				4	M	Oral cavity	Biopsy, DIF	NR				Dap, Pred, Aza	88 months	NR			
13	Kulkarni et al. 2021 [11]	Case report	1	6	F	Skin, oral cavity	IIF	NR	Oral Pred at 2 mg/kg/day	Incomplete remission, side effects of CRs	Two infusions of 500 mg each at an interval of 15 days	Top-up IVIG in a dose of 400 mg/kg was tried but the child developed an infusion reaction (fever, chills, headache, and abdominal pain)	1 year	No	Complete remission off therapy	None	RTX is a feasible therapeutic option and needs further studies for validation of its use in children.
14	Mamelak et al. 2007 [9]	Case series	2	16	F	Skin, oral cavity	Biopsy and IF	27 months	IM CRs initially, followed by oral Pred and MMF. Later, Aza 200 mg, and IVIG	Hip avascular necrosis at the hip, recurrence of lesions despite multiple Pred and IVIG	375 mg/m^2^ weekly for 4 weeks, then another cycle 6 months later	6 cycles of plasmapheresis were given over 2 weeks	6 months	Yes	Complete remission on Aza 200 mg daily maintenance therapy	NR	RTX is efficacious in pediatric PV.
				16	F	Hand, abdomen, lower back, and oral cavity	Biopsy and IF		Pred 1 mg/kg, MMF 40 mg/kg daily (divided dose), 6 cycles of plasmapheresis	Disease progression despite treatment	375 mg/m^2^ weekly for 4 weeks	narcotics for pain control, adjuvant treatment with IVIG 2 g/kg. MMF was discontinued and Aza 250 mg daily initiated	6 months	Yes	Complete remission	NR	
15	Salman et al. 2017 [31]	Retrospective analysis	2	14	M	Skin and oral mucosa	Biopsy and IF	1 month	RTX concurrent therapy with other agents	Multiple therapy failure	4 cycles of RTX	MP, Dap, IVIG, Aza	24 months	No	Complete remission on treatment	Dental abscess	Good prognosis observed following RTX therapy.
				16	M	Oral cavity	Biopsy and IF	2 months			2 cycles of RTX	MP, Dap, IVIG, MMF, Aza	44 months	No	Complete remission off treatment	None	
16	Srivastava et al. 2017 [32]	Case report	1	14	M	Skin and oral cavity	IIF	6 months	Topical TCA and topical anesthetics	Severely progressing lesions despite treatment	IV RTX 500 mg weekly for 1 month	AV gel	Patient was on follow-up	NR	Oral lesions healed rapidly, and the skin lesions healed with pigmentation	NR	CRs are the first-line therapy for PV. RTX in refractory cases.
17	Vaquez et al. 2023 [33]	Case report	1	14	F	Skin and oral mucosa	Biopsy	2 months	IV/oral CRs and MMF 500 mg daily. IVIG treatment	Mild relief and CR adverse effects	IV RTX 4 cycles of 375 mg/m^2^, 1 week apart	Oral CRs	18 months	No	Rapid clinical remission in 2 weeks of infusion. Complete remission off therapy	None	RTX therapy can be used for recalcitrant lesions.
18	Vinay et al. 2014 [34]	Retrospective analysis	7	9	M	Mucosal and skin lesions	Biopsy, DIF, IIF, ELISA	6 months	Aza, CRs, DMP therapy	Refractory to CT, severe disease	2 doses of 375 mg/m^2^ of body surface area	CRs	36 months	Yes	Complete remission off treatment	Angioedema	No long-term or serious complications observed.
				11	M			12 months	Aza, CRs, DMP therapy	Severe disease	2 doses of 375 mg/m^2^ of body surface	CRs	8 months	No	Complete remission off treatment	Infusion reaction	
				17	M			12 months	Aza, CRs, DMP therapy, MMF	Refractory to CT, severe disease	2 doses of 500 mg	Aza, CRs	19 months	Yes	Complete remission off treatment	None	
				17	M			84 months	CRs	Refractory to CT therapy, CRs adverse effects	2 doses of 500 mg	Aza, CRs	18 months	Yes	Complete remission on treatment	Infusion reaction	
				17	F			36 months	CRs	Severe disease	2 doses of 500 mg	CRs	17 months	No	Complete remission off treatment	None	
				13	F			12 months	Aza, CRs	Refractory to conventional therapy	2 doses of 500 mg	CRs	14 months	No	Complete remission off treatment	URT infection	
				12	M			30 months	Aza, CRs	Refractory to CT	2 doses of 500 mg	CRs	12 months	Yes	Control of disease activity	Angioedema	

A total of 46 juvenile or childhood PV patients in the pediatric and adolescent age groups were reviewed in the present study, out of which 24 were males and 22 were females. The youngest patient was 18 months old, whereas the upper age limit was 17 years. Nine case reports, two case series, five retrospective studies, one prospective, and one open-labeled pilot study were included in this systematic review. Widely distributed lesions with the involvement of both cutaneous and mucous membranes were observed in the pediatric PV. The common sites reported in this review were the face, trunk, extremities, scalp, and oral mucosa.

Before RTX therapy, various drugs were instituted in pediatric PV patients including systemic corticosteroids such as prednisolone and dexamethasone as the primary treatment modality. One study also reported the use of the topical corticosteroid triamcinolone acetonide [32]. Other immunosuppressants such as azathioprine [9,28,30,31], cyclosporine [24], mycophenolate mofetil [9,30,31], and intravenous immunoglobulins (IVIG) [9,11,31] were also administered.

A commonly followed RTX regimen (lymphoma regimen), based on body surface area where 300/375/500 mg/m² of RTX was administered as two infusions 15-30 days apart or four infusions were given weekly. RTX was supplemented with additional treatment modalities in most cases, which primarily involved corticosteroids, azathioprine, or IVIG. Additional infusions of RTX itself were also given in some cases [19, 28].

Study Outcomes

Outcomes or endpoints were described in terms of remission, either complete or partial. Nearly all studies reported complete remission on treatment (n=20; 43.4%), off treatment (n=20; 43.4%), or complete remission without the status of ongoing treatment (n=3; 6.5%) or partial remission off treatment (n=2; 4.3%) at the final follow-up visit (n=45/46; 97.8%). Control of disease activity was reported in one case [33]. Relapses or flares were observed in 18 cases (39.1%) either minor or major, during initial follow-ups. No relapse was noted in 22 cases (47.8%) whereas six cases did not report this information. The longest follow-up of 16 years was reported in the study by Fuertes et al. [23], whereas the minimum follow-up period was six months [9]. Minimal adverse effects were noted following RTX therapy. A few side effects that were reported include infusion reactions, Herpes Zoster infection, angioedema, chills, fever, tachycardia, urticaria, upper respiratory tract infections (URTI), and dental abscess [24,25,27,28,31,34].

Quality Assessment

**Figure 2:**
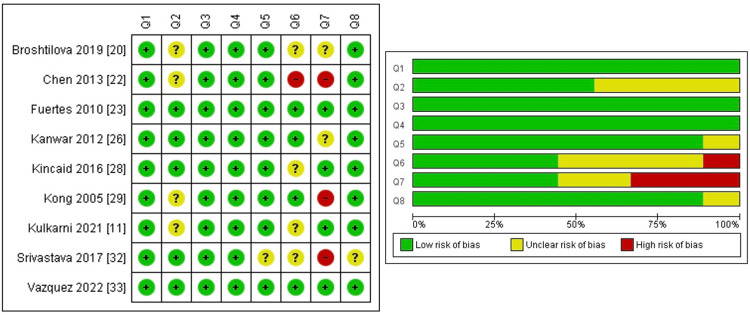
Risk of bias: summary and graph assessed through JBI critical appraisal checklist for case reports. JBI: Joanna Briggs Institute.

The overall risk of bias for the case reports as assessed through the JBI Critical Appraisal Checklist was found to be low. Some concerns were about the reporting of history as a timeline and missing data on adverse events across some studies (Figure 2).

**Figure 3:**
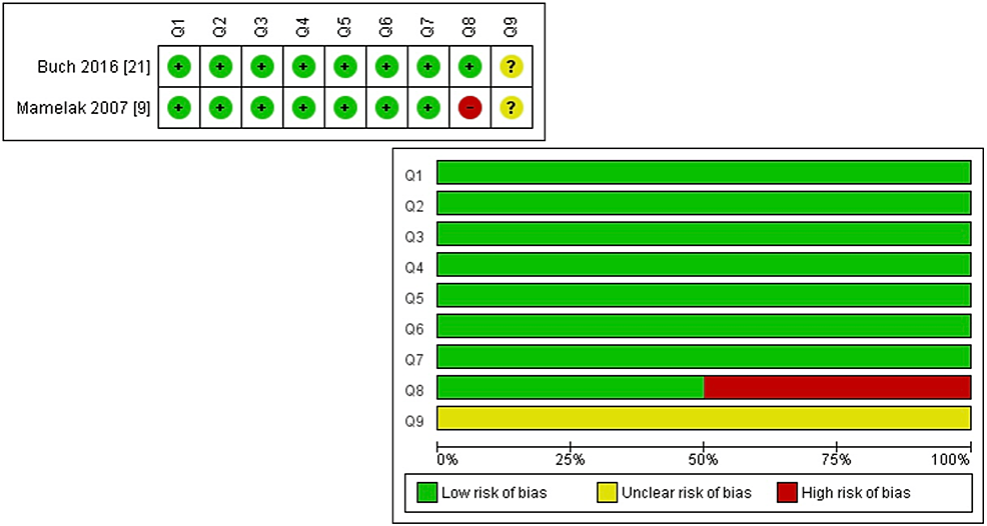
Risk of bias: summary and graph assessed through JBI critical appraisal checklist for case series. JBI: Joanna Briggs Institute.

Regarding case series, there were unclear risks regarding the reporting of the presenting site(s)/clinic(s) demographics (Figure 3).

**Figure 4:**
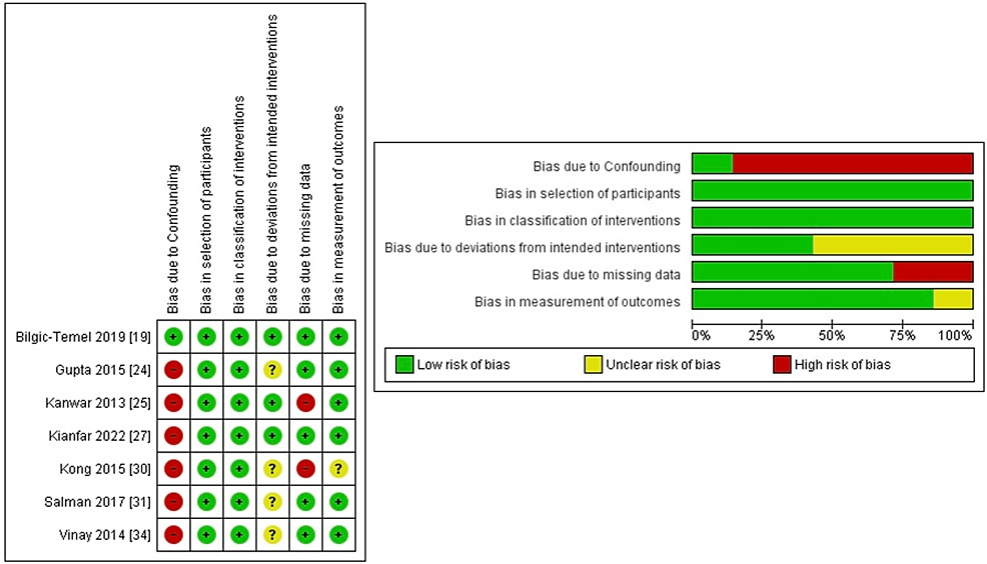
Risk of bias: summary and graph observational/non-randomized studies of intervention assessed through Cochrane ROBINS-I tool.

For observational and non-randomized interventional studies, a high risk of bias was noted in the domain of bias due to confounding, possibly due to alternative treatment regimens administered alongside RTX, which could potentially alter the outcome. There were also a few concerns due to missing data concerning occurrences of relapse and remission. The quality assessment for this group of studies is summarized in Figure 4.

Discussion

Autoimmune bullous diseases in childhood comprise a collection of rare blistering disorders affecting the skin and mucous membranes. Corticosteroids serve as the primary treatment; however, due to the susceptibility of the pediatric population to their serious adverse effects, there is a pressing need for safer alternatives, particularly in cases that are recalcitrant and severe [35]. RTX, a monoclonal anti-CD20 antibody recently approved by the FDA for moderate to severe PV in adults, emerges as a promising alternative [36]. However, its efficacy, safety, and cost-effectiveness need evaluation.

This comprehensive review synthesizes evidence from 18 studies, including clinical data from 46 patients where RTX was administered for the treatment of pediatric PV, systematically examining its outcomes, adverse events, and further implications.

In this review, nearly all cases of childhood and juvenile PV (n=45; 97.8%) achieved complete or partial remission following treatment with RTX during final follow-up intervals. Moreover, a majority of cases (n=22; 47.8%) reported no relapse, with minor adverse events documented in the RTX treatment group. The overall prognosis appeared favorable, demonstrating promise in these preliminary investigations.

However, the clinical efficacy of RTX remains uncertain due to various confounding factors observed in the reported cases. These confounding factors include prior treatment with glucocorticoids and other steroid-sparing agents, concurrent therapy with corticosteroids, cyclophosphamide, azathioprine, methotrexate, and intravenous immunoglobulin (IVIG). Additionally, variability in drug dosage and treatment duration across studies, as well as the absence of randomization or controlled trials, further complicates the evaluation of RTX efficacy.

Across the studies reviewed, the indications for RTX therapy were treatment failure in cases of refractory or recalcitrant PV lesions, or relapses despite prolonged treatment with multiple immunosuppressants. According to a consensus statement, a case of PV is considered refractory if previous lesions continue to spread, new lesions develop, or established lesions fail to heal after three weeks of therapy with 1.5 mg/kg/day of prednisolone or its equivalent, along with concurrent use of cyclophosphamide at 2 mg/kg/day or azathioprine at 2.5 mg/kg/day for 12 weeks [16].

Long-term corticosteroid therapy has been associated with adverse drug reactions, prompting the exploration of alternative unconventional therapies with fewer potential side effects. Common adverse effects observed in the pediatric and adolescent age group include Cushing syndrome, stunted growth and development, elevated liver function tests, hypertrichosis, and edema. Furthermore, severe childhood pemphigus also warranted consideration for first-line treatment with RTX therapy [27].

The standard route of administration for RTX is intravenous infusion, which carries the risk of infusion-related reactions, as noted in a few reported cases. However, a subcutaneous route has been suggested to enhance ease of administration, increase patient convenience, and ensure cost-effectiveness [37].

The duration of treatment typically spans two to three years, potentially exacerbating long-term effects in the pediatric age group. Nevertheless, compared to prolonged corticosteroid administration, steroid-sparing agents like RTX offer a safer alternative. Other steroid-sparing agents such as azathioprine [9,28,30,31], mycophenolate mofetil [9,30,31], IVIG [9,11,30], dapsone [30], methotrexate [30], and cyclophosphamide [24] were also used. Azathioprine was used for 14 and 17 months [30], mycophenolate mofetil for seven months [30], dapsone for 12 months [30], and 10-13 cycles of IVIG [30]. Cataract, osteopenia, lymphopenia, and cushingoid effects were the major adverse effects observed with azathioprine, mycophenolate mofetil, and IVIG [30], whereas dapsone usage resulted in hemolytic anemia [30,31].

In the present review, no long-term side effects on growth and development were noted following RTX therapy. While relatively milder adverse events were observed with RTX, it is worth noting that one included study reported the death of a patient, although in the adult population [25].

A comprehensive diagnosis of pemphigus is based on four criteria: (a) clinical presentation, (b) histopathologic examination of a lesional biopsy, (c) direct immunofluorescence (DIF) examination of a perilesional skin or mucosal biopsy, and (d) serological detection of autoantibodies against epithelial cell surfaces by indirect immunofluorescence (IIF) and/or enzyme-linked immunosorbent assay (ELISA Dsg1 and Dsg3). Serological detection and differentiation of circulating autoantibodies by enzyme-linked immunosorbent assays (ELISA) form the cornerstone of pemphigus diagnostics [38].

Diagnostic tests for PV do not differ between adult patients and children, as both have similar clinical, histological, and immunological features [39].

This systematic review encompassed case reports, case series, and retrospective studies, all lacking an established control group, resulting in a low body of clinical evidence. Moreover, significant heterogeneity was evident among the included studies, characterized by variable follow-up periods, indications for RTX administration, concurrent therapies alongside RTX, and varying dosage and frequency cycles of RTX. Meta-analysis could not be performed due to subjective outcome reporting and substantial heterogeneity. Standardized outcome reporting is recommended to enhance homogeneity for future meta-analyses. Additional limitations observed in the current systematic review included potential publication bias towards positive outcomes, limited generalizability of findings due to the rarity of pediatric pemphigus cases, and the retrospective nature of most included studies, which may introduce recall and selection biases.

## Conclusions

The efficacy of RTX treatment for pediatric PV is promising but lacks strong evidence due to limited studies. RTX demonstrates a high rate of complete or partial remission in cases of refractory PV among pediatric patients and offers a safer alternative to glucocorticoids, which carry risks of long-term adverse effects like growth retardation.

Despite its potential, the use of RTX in pediatric patients remains questionable due to the limited evidence base and lack of controlled studies in this age group.

Further testing and comparison of clinical parameters are needed to establish a standardized treatment protocol for RTX in pediatric PV, including optimal dosage, frequency, duration of treatment cycles, and maintenance therapy length.

## Appendices

**Table 3:** Risk of bias for case reports.

Sno.	Questions	Description
1	Q1	Were the patient’s demographic characteristics clearly described?
2	Q2	Was the patient’s history clearly described and presented as a timeline?
3	Q3	Was the current clinical condition of the patient on presentation clearly described?
4	Q4	Were there diagnostic tests or assessment methods, and were the results clearly described?
5	Q5	Was the intervention(s) or treatment procedure(s) clearly described?
6	Q6	Was the post-intervention clinical condition clearly described?
7	Q7	Were adverse events (harms) or unanticipated events identified and described?
8	Q8	Does the case report provide takeaway lessons?

**Table 4:** Risk of bias for case series.

Sno.	Questions	Description
1	Q1	Were there clear criteria for inclusion in the case series?
2	Q2	Was the condition measured in a standard, reliable way for all participants included in the case series?
3	Q3	Were valid methods used for the identification of the condition for all participants included in the case series?
4	Q4	Did the case series have consecutive inclusion of participants?
5	Q5	Did the case series have complete inclusion of participants?
6	Q6	Was there clear reporting of the demographics of the participants in the study?
7	Q7	Was there clear reporting of clinical information of the participants?
8	Q8	Were the outcomes or follow-up results of cases reported?
9	Q9	Was there clear reporting of the presenting site(s)/clinic(s) demographic information?
